# Design, Synthesis, Biological Evaluation, and Preliminary Mechanistic Study of a Novel Mitochondrial-Targeted Xanthone

**DOI:** 10.3390/molecules28031016

**Published:** 2023-01-19

**Authors:** Sibei Wang, Qi Zhang, Maoqin Peng, Jing Xu, Yuanqiang Guo

**Affiliations:** 1 State Key Laboratory of Medicinal Chemical Biology, College of Pharmacy, and Tianjin Key Laboratory of Molecular Drug Research, Nankai University, Tianjin 300350, China; 2 Key Laboratory of Tropical Medicinal Resource Chemistry of Ministry of Education, Hainan Normal University, Haikou 571158, China

**Keywords:** *α*-mangostin, triphenylphosphonium, mitochondrial targeting, antitumor activity, apoptosis, zebrafish

## Abstract

*α*-Mangostin, a natural xanthone, was found to have anticancer effects, but these effects are not sufficient to be effective. To increase anticancer potential and selectivity, a triphenylphosphonium cation moiety (TPP) was introduced to *α*-mangostin to specifically target cancer cell mitochondria. Compared to the parent compound, the cytotoxicity of the synthesized compound **1b** increased by one order of magnitude. Mechanistic analysis revealed that the anti-tumor effects were involved in the mitochondrial apoptotic pathway by prompting apoptosis and arresting the cell cycle at the G0/G1 phase, increasing the production of reactive oxygen species (ROS), and reducing mitochondrial membrane potential (Δψ_m_). More notably, the antitumor activity of compound **1b** was further confirmed by zebrafish models, which remarkably inhibited cancer cell proliferation and migration, as well as zebrafish angiogenesis. Taken together, our results for the first time indicated that TPP-linked **1b** could lead to the development of new mitochondrion-targeting antitumor agents.

## 1. Introduction

Traditional chemotherapy drugs are used to treat many types of cancers. Though chemotherapy treatment is an effective method to combat cancer, it also faces limitations, such as cytotoxicity to normal cells, poor water solubility, poor bioavailability, and the emergency of multi-drug resistance [1]. In recent years, targeted therapeutic drugs have emerged and replaced some conventional chemotherapy agents as the mainstream treatments for cancer due to their advantages in safety and efficacy [2]. Mitochondria play a significant role in cancer through energy production and macromolecular synthesis, which can produce more metabolic precursors for macromolecules, such as RNA, DNA, proteins, and lipids, to meet the requirement of the rapid tumor cell proliferation. Because of the vital role of mitochondria in tumor progression, mitochondrion-targeted antitumor therapy has become a hot topic in recent years [3]. Mitochondrion-targeted antitumor agents can trigger mitochondrial dysfunction via different mechanisms, leading to cancer cell death.

Mitochondria are an organelle found in eukaryotic cells and have vital functions in energy conversion. Various physiological activities, such as energy metabolism and electron transfer, are closely related to mitochondria. In addition, mitochondria are regarded as the central control point of apoptosis triggered by a variety of stimuli [4,5]. Mitochondria have a double-membrane system that includes an inner and an outer membrane. The difference in electric potential between the two membranes is called mitochondrial membrane potential (Δψ_m_), which is much higher in tumor cells than that in normal cells [3]. Therefore, using this feature, some positively charged drug molecules or agents such as rhodamine 123, dequalinium, guanidinium, and triphenylphosphonium (TPP) cations can be driven by Δψ_m_ to accumulate in mitochondria and achieve targeted antitumor effects able to destroy cancer cells [6,7,8].

Mitochondrion-targeted tumor therapy has been widely studied in recent years. In 2017, a review summarized the great progress made in the antitumor effects of mitochondrion-targeted therapeutics [3]. In this review, numerous TPP-linked mitochondrion-targeted drugs, including mito-resveratrol, mito-quercetin, and mito-curcumin, have been reported to offer enhanced anticancer effects. Natural plant polyphenols such as resveratrol, quercetin, and curcumin, continue to be the subject of extensive investigations due to their promising anticancer properties and relatively low toxicity. Thus, hydroxy or carboxyl groups in these bioactive natural products can be linked with mitochondrion-targeted TPP to improve antitumor activities [6,7,8].

Xanthones are a type of phenolic compounds with hydroxy groups showing extensive biological and pharmacological activities [9]. Mangosteen (*Garcinia mangostana*), a tropical fruit tree, is an abundant source of xanthones and derivatives. *α*-Mangostin was found to be rich in mangosteen, which has diverse pharmacological activities including antioxidant, antidiabetic, anti-inflammatory, and anticancer effects [10,11,12]. In our previous study, the xanthone *α*-mangostin (**1**) was isolated from the fruit hull of *Garcinia mangostana*. During our screening for anticancer active compounds, *α*-mangostin showed moderate antitumor effects. To obtain more active compounds, structural modification targeting mitochondria was designed, which yielded TPP-linked *α*-mangostin (**1b**). Moreover, in vitro and in vivo anticancer activity and the preliminary mechanism of action of **1b** were studied.

## 2. Results

### 2.1. Molecular Design and Chemical Synthesis

**Scheme 1:**
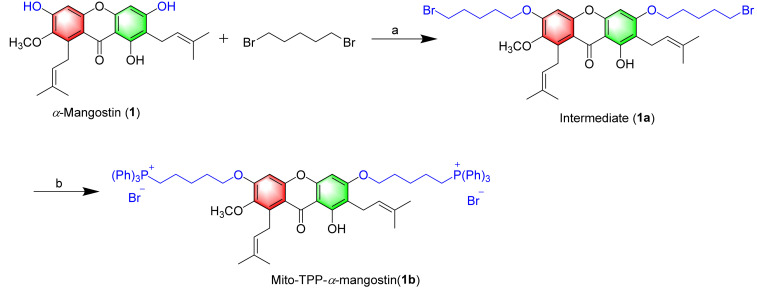
Synthesis of target derivative **1b**. Reaction conditions: (**a**) K_2_CO_3_, 1,5-dibromopentane, acetone, 65 °C, reflux 6 h and (**b**) TPP (Triphenylphosphine), toluene, 80 °C, reflux 12 h.

One TPP-linked *α*-mangostin derivative (**1b**) was designed and synthesized as shown in Scheme 1. Firstly, an intermediate (**1a**) was obtained through etherification at the C-3 and C-6 hydroxy group sites of **1** with 1,5-dibromopentane linkers, resulting in a 60% yield. Then, the intermediate **1a** reacted with triphenylphosphine to obtain a 47% yield of the desired end-product **1b**. The structure of **1b** was confirmed using high-resolution mass, ^1^H NMR, and ^13^C NMR spectra (Supplementary Materials).

### 2.2. In Vitro Biological Activity

To verify whether the antitumor activity of **1b** was improved compared to the parent compound **1**, in vitro cytotoxic effects of these two compounds were evaluated against four tumor cell lines (human lung adenocarcinoma cells A549, human chronic myeloid leukemia cells K562, human liver cancer cells HepG2, and human cervical cancer cells Hela). Etoposide was used as a positive control. The findings are presented in Table 1. Compound **1b** showed much better cytotoxicity than both parent compound **1** and etoposide against all four cancer cell lines, and the cytotoxicity of **1b** was about 5–8 times stronger than that of its parent compound **1**. Clearly, the modification of parent compound **1** was able to significantly increase the compound’s ability to kill cancer cells. Because **1b** had the most pronounced effects on A549 cells, subsequent analyses on the mechanism of action of **1b** were performed in A549 cells.

### 2.3. Apoptosis Effects Induced by Compound ***1b*** in A549 Cells

**Figure 1:**
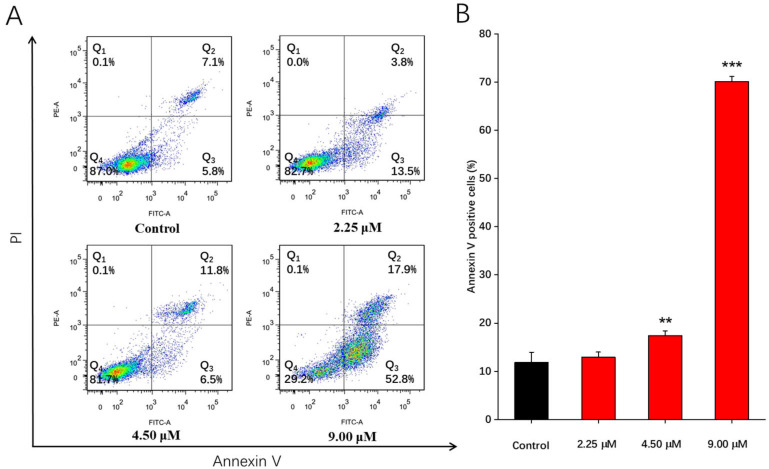
Compound **1b**-induced apoptosis on A549 cells. A549 cells were treated with 2.25, 4.50, and 9.00 μM of compound **1b** for 48 h. DMSO was used as a negative control. Apoptotic levels were assessed using Annexin V FITC/PI double staining and flow cytometry. (**A**) Flow cytometric analysis of A549 cell distribution after treatment with different concentrations of compound **1b**. (**B**) Related histogram for apoptotic cells are expressed as the means ± SD (*n* = 3). ** *p* < 0.01, *** *p* < 0.001 vs. the control group.

To detect whether compound **1b** could induce apoptosis in A549 cells, the Annexin V FITC/PI double staining technique was applied, and cell death was assessed via flow cytometric analysis [8]. A549 cells were challenged with compound **1b** (2.25, 4.50, and 9.00 μM) for 48 h. The subsequent results of cell apoptosis are presented in Figure 1. A549 cells treated with compound **1b** showed an increased total percentage of apoptotic cells from 12.9% in control cells to 17.3%, 18.3%, and 70.7% at 2.25, 4.50, and 9.00 μM, respectively. These findings demonstrated that 9.00 μM of compound **1b** could most significantly induce the apoptosis of A549 cells compared to the control group.

### 2.4. Arrest Effects of Compound ***1b*** on A549 Cell Cycle

**Figure 2:**
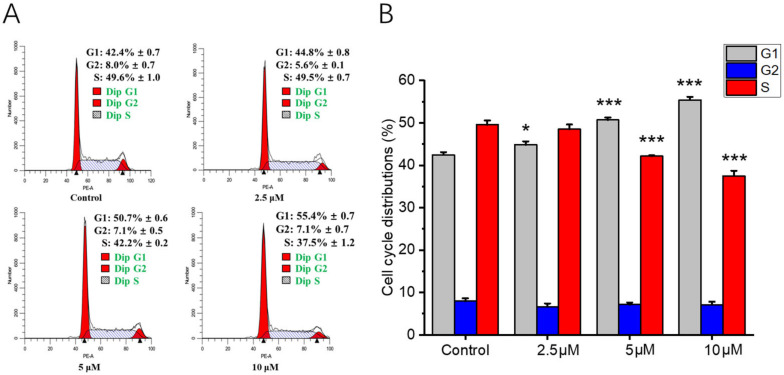
Arrest effects of compound **1b** on cell cycle distribution in A549 cells. A549 cells were treated with 2.5, 5, and 10 μM of compound **1b** for 48 h. DMSO was used as a negative control. **(A**) The cell cycle ratio of A549 cells was detected using a cell cycle staining kit, and the cell cycle distribution was analyzed using flow cytometry. (**B)** Related histogram of cell cycle phases distribution, expressed as the mean ± SD (*n* = 3). * *p* < 0.05, *** *p* < 0.001 vs. the control group.

Apoptosis, or programmed cell death, is inextricably linked to cell cycle progression. To evaluate the effects of compound **1b** on cell cycle distribution, A549 cells were cultured with compound **1b** (2.5, 5, and 10 μM) for 48 h. Following PI staining, cells were measured via flow cytometry [13,14]. As shown in Figure 2, the proportion of A549 cells in the G1 phase increased from 42.4% (control) to 44.8% (2.5 μM), 50.7% (5 μM), and 55.4% (10 μM) as the concentration of compound **1b** increased from 2.5 to 10 μM. The results revealed that compound **1b** could induce G0/G1 cell cycle arrest.

### 2.5. The Increase in Intracellular Reactive Oxygen Species (ROS) Production Stimulated by ***1b***

**Figure 3:**
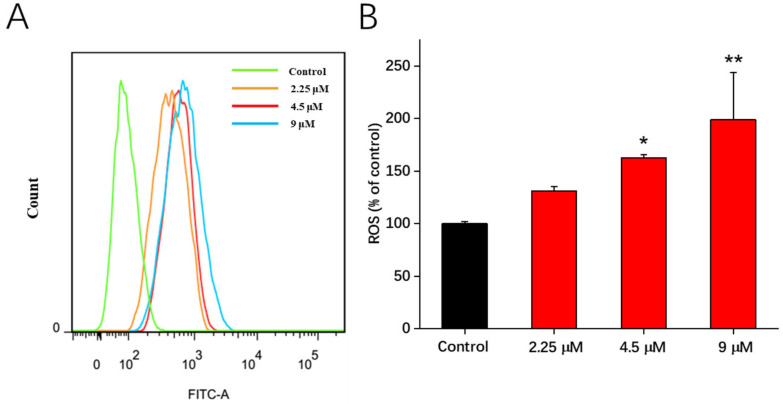
Effects of compound **1b** on ROS generation in A549 cells. Cells were treated with compound **1b** at the tested concentrations for 48 h. (**A**) ROS production in A549 cells was measured via flow cytometric analysis using DCFH-DA staining. (**B**) Related histograms for ROS levels are presented as the means ± SD (*n* = 3). * *p* < 0.05, ** *p* < 0.01 vs. the control group.

One of the most important mediators of apoptotic signaling is ROS production [15,16]. To determine the ROS in A549 cells, the DCFH-DA method was employed [15,17]. After 48 h of treatment with compound **1b** (2.25, 4.5, and 9 μM), a generation of more ROS was observed in our investigation. As the concentrations of compound **1b** increased, the content of the ROS increased significantly in comparison to the control group (Figure 3). These findings indicated that the ROS production was critical in the apoptosis triggered by compound **1b**.

### 2.6. Effects of Compound ***1b*** on the Mitochondrial Membrane Potential (Δψ_m_)

**Figure 4:**
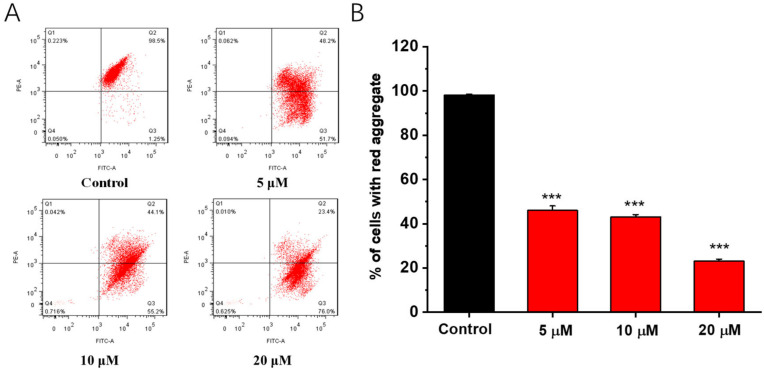
Effects of compound **1b** on mitochondrial membrane potential (Δψ_m_) in A549 cells. (**A**) Cells were treated with compound **1b** at the tested concentrations for 48 h and stained with JC-1 dye. Δψ_m_ was analyzed via flow cytometry. (**B**) The quantification of % of cells with red aggregates. The values are presented as the means ± SD (*n* = 3). *** *p* < 0.001 vs. control group.

Mitochondrial membrane potential is crucial for maintaining normal cellular physiological functions, and the reduction of Δψ_m_ is a crucial factor in both extrinsic and intrinsic apoptosis [18,19]. To investigate the effects of A549 tumor cells on the mitochondrial membrane potential, cancer cells were stained with JC-1 after 48 h of treatment with compound **1b** (5, 10, and 20 μM) and examined using flow cytometry [8]. As shown in Figure 4, compound **1b** significantly reduced the mitochondrial membrane potential, and the high concentration of compound **1b** in the 20 μM group reduced the mitochondrial membrane potential of A549 cells by more than 70%.

### 2.7. Effects on the Expression of Mitochondrial Pathway Proteins

A decrease in mitochondrial membrane potential is an early sign of apoptosis. As shown in Figure 4, compound **1b** significantly reduced the mitochondrial membrane potential. To further explore whether the effects of compound **1b** on the mitochondrial membrane potential could promote apoptosis through the mitochondrial pathway, the technique of Western blotting was utilized. Mitochondrial outer membrane permeabilization (MOMP) is the main event in the mitochondrion-mediated pathway of apoptosis. Upon oxidative stress, the binding of mitochondria to the Bcl-2 family proteins Bax and Bak induces an increase in the MOMP, and the mitochondrial permeability transition pore (PTP) opens. Then, after reduction of the mitochondrial membrane potential, cytochrome c enters the cytosol from the intermembrane space (IMS) of the mitochondria and binds to apoptotic protease activator (Apaf-1) to induce the formation of the apoptosome, resulting in caspase-9 and caspase-3/7 cleavage and activation with a caspase-dependent apoptotic signaling cascade response [20].

**Figure 5:**
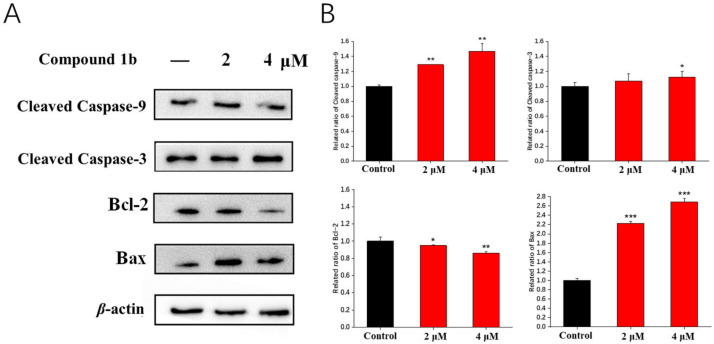
Effects of compound **1b** on the expression of four apoptosis-related proteins. Cells were treated with compound **1b** at the indicated concentrations for 48 h. (**A**) Representative images of Western blotting analysis of cleaved caspase-9, cleaved caspase-3, Bcl-2, and Bax. (**B**) Quantitative analyses of Western blotting are presented as the means ± SD (*n* = 3). * *p* < 0.05, ** *p* < 0.01, *** *p* < 0.001 vs. the control group.

To further determine the mechanism of action of compound **1b** in triggering apoptosis on A549 cells, we examined the expression of four apoptosis-related proteins, cleaved caspase-9, cleaved caspase-3, Bcl-2, and Bax, using a Western blot assay. The data indicated that compound **1b** up-regulated the expression of Bax and down-regulated the expression of Bcl-2, which triggered a cascade effect that increased the expression of cleaved caspase-9 and cleaved caspase-3 (Figure 5). Therefore, we demonstrated that compound **1b** could induce the apoptosis of A549 cells through the mitochondrial apoptotic pathway.

### 2.8. In Vivo Antitumor Effects of Compound ***1b*** in Zebrafish Xenografts

**Figure 6:**
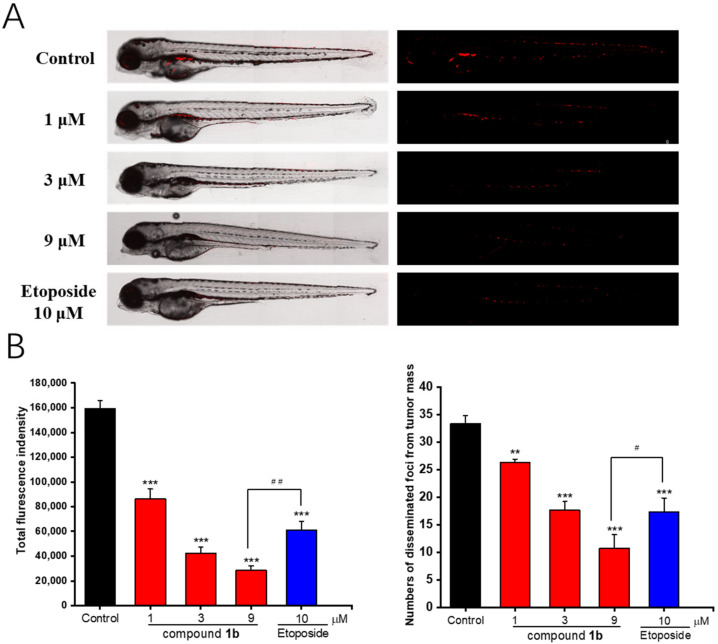
In vivo antitumor effects of compound **1b** in zebrafish xenografts. CM-DiI-stained A549 cells were transplanted into two dpf zebrafish embryos via microinjection. (**A**) Representative images of the relative intensity and distribution of the red fluorescence using confocal microscopy after testing the concentration of compound **1b** administration (1, 3, and 9 μM) for 48 h. (**B**) Histogram of fluorescence intensities and quantification of the fluorescent area of the compound **1b**-treated tumor xenografts. Results are presented as the means ± SD (*n* = 3). ** *p* < 0.01, *** *p* < 0.001 vs. the control group. ^#^ *p* < 0.05, ^##^ *p* < 0.01 vs. the positive control etoposide.

Finally, to determine mitochondrion-targeting antitumor agents, an in vivo activity evaluation of compound **1b** was carried out. The in vitro study showed that the cytotoxicity of **1b** was about 5–8 times stronger than that of its parent compound **1**. Therefore, we used 48 hpf healthy wild zebrafish AB strain as an experimental animal model to further investigate the in vivo antitumor effects of the different concentrations of compound **1b** (1, 3, and 9 μM) and a positive control etoposide (10 μM) in a zebrafish xenotransplanted with A549 lung cancer cells. Based on the results shown in Figure 6, the intensity of the red fluorescence in zebrafish was stronger in the blank group than that in the drug-treated groups. Additionally, the intensity of the red fluorescence in the dosing groups gradually decreased with an increasing concentration of compound **1b**. Compound **1b** was able to inhibit more than 80% of tumor proliferation relative to the control group at the maximum dosing concentration of 9 μM. Moreover, a significant difference was found between the **1b**-treated group (9 μM) and the etoposide-treated group (10 μM), suggesting that 9 μM of the **1b**-treated group offered a much better in vivo antitumor activity than the positive control etoposide. Therefore, the mitochondrial-targeted modification significantly enhanced the cytotoxicity of compound **1**, and the modified compound **1b** had better in vivo activity than the positive control etoposide.

### 2.9. In Vivo Antiangiogenic Activity of Compound ***1b*** in a Transgenic Zebrafish Model

**Figure 7:**
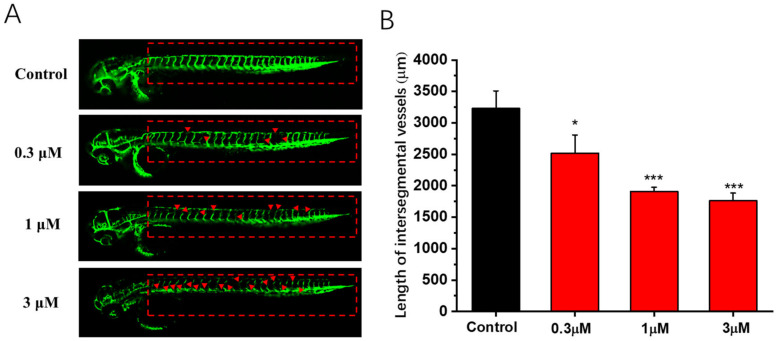
Antiangiogenesis activity of compound **1b** in the transgenic zebrafish model. (**A**) Representative images of zebrafish embryos from transgenic zebrafish *Tg* (*fli1:EGFP*) treated with the vehicle and different concentrations of compound **1b** (0.3, 1, and 3 μM) at 48 hpf. The absence and breakage of ISVs are indicated by red arrows. (**B**) Average total lengths of the ISVs of zebrafish after treatment with various concentrations of compound **1b**. Results are presented as the means ± SD (*n* = 3). * *p* < 0.05, *** *p* < 0.001 vs. the control group.

Angiogenesis is a critical step in the formation of new blood vessels, which is a key contributor to tumor growth and metastasis [21,22,23]. Mitochondria play a crucial role in controlling angiogenesis by regulating the proliferation, migration, and survival of endothelial cells, which form the inner lining of blood vessels. Hence, we used a transgenic *Tg* (*fli1:EGFP*) zebrafish model to explore the antiangiogenic activity of compound **1b**. Zebrafish embryos were incubated with compound **1b** at the indicated concentrations (0.3, 1, and 3 μM) for 48 h, and the formation of intersegmental vessels (ISVs) was analyzed and quantified by the Image J software. As depicted in Figure 7, the zebrafish embryos in the blank control group had a normal gross morphological development, and ISVs were intact with a clear and complete expression of green fluorescence. However, the ISVs of zebrafish embryos were substantially damaged or lost following an exposure to various concentrations of compound **1b**. Additionally, the total lengths of ISVs in zebrafish embryos were quantified and showed a decrease with increasing concentrations of compound **1b** (2518.39 ± 289.92 μm at 0.3 μM, 1910.59 ± 64.56 μm at 1 μM, and 1764.22 ± 120.47 μm at 3 μM). The results showed that compound **1b** could inhibit ISV formation in zebrafish embryos.

## 3. Discussion

In the development of effective anticancer agents, mitochondria are acknowledged to be an important drug target [3]. The triphenylphosphonium-based modification of drugs to facilitate mitochondrial targeting is not a novel idea, as there is a wealth of literature on the good biological effects of small molecules containing TPP [24,25,26,27,28,29,30,31]. The hydroxy and carboxyl groups of natural products are some activating groups that are often used to link pharmacophores such as TPP [6,7,8]. *α*-Mangostin containing phenolic hydroxy groups was isolated from the fruit hull of *Garcinia mangostana* in our previous study. During our extensive screening for natural antitumor agents, *α*-mangostin (**1**) showed moderate antitumor activities. Thus, to discover mitochondrion-targeting antitumor agents, a structural modification of compound **1** was designed, and this modified compound was used to synthesize the corresponding mitochondrion-targeted compound (**1b**) through conjugation with the triphenylphosphonium cation (TTP) moiety via aliphatic 1,5-dibromopentane linkers.

Considering the limited availability of natural-sourced *α*-mangostin (**1**), firstly, an attempt was made to link 1,5-dibromopentane with triphenylphosphine, followed by substitution to an ether reaction with *α*-mangostin. However, it was found that the reaction between the hydrophilic quaternary phosphine salts obtained from the first reaction of 1,5-dibromopentane with triphenylphosphine and the hydrophobic *α*-mangostin was not efficient due to the choice of reaction solvents. Reaction solvents such as acetone, acetonitrile, tetrahydrofuran, and toluene were employed, but the reaction was still inefficient. Moreover, considering that the final product was triphenylphosphine salts, and the precursor obtained in the first step was also quaternary phosphine salts, it was difficult to distinguish and isolate the final product from the precursor. Therefore, the design strategy was changed, and a successful design route was obtained, as shown in Scheme 1.

To design a new drug that specifically targets mitochondria, we used compound **1** as the starting material to synthesize the TTP-linked derivative **1b**. In vitro antitumor screening indicated that the antitumor activities of compound **1b** against four cancer cell lines were about one order of magnitude more cytotoxic than the activities of compound **1**. To further investigate the antitumor mechanism of compound **1b**, additional pharmacological experiments were performed both in vivo and in vitro.

Countless studies have clearly demonstrated that the mitochondrial apoptotic pathway is connected with cell cycle arrest and the excessive production of ROS, which subsequently triggers a decrease in mitochondrial membrane potential and the release of cytochrome c and other cofactors from the mitochondria, inducing subsequent caspase activation regulated by Bcl-2 family proteins [4,5,20]. Our subsequent preliminary mechanistic investigation demonstrated that **1b** exerts significant cytotoxic effects by inducing apoptosis and arresting the A549 cell cycle at the G0/G1 phase, as shown in Figure 1 and Figure 2. Furthermore, **1b** was found to induce ROS production, and reduce mitochondrial membrane potential, as illustrated in Figure 3 and Figure 4. It has been extensively reported that Bcl-2 family proteins are key regulators in the mitochondrial apoptotic pathway. Additionally, the level of Bcl2-related protein expression was assessed. As a result, **1b** up-regulated the expression of Bax, cleaved caspase-9, and cleaved caspase-3 and down-regulated the expression of Bcl-2 (Figure 5). These findings demonstrated that **1b** could induce apoptosis by triggering the mitochondrial apoptotic pathway.

Notably, zebrafish are considered robust and reliable animal models. Hence, zebrafish xenograft models were employed to evaluate the effects of **1b** on the proliferation and metastasis of A549 cells in vivo. Furthermore, angiogenesis plays a pivotal role in cancer progression because it enables the delivery of growth factors, nutrients, and oxygen, as well as the dissemination of tumor cells to distant organs [21,22]. Thus, inhibiting angiogenesis is a crucial strategy for the prevention of various solid tumors [23]. To investigate if **1b** exerted inhibitory activities on zebrafish angiogenesis, we utilized the transgenic *Tg* (*fli1:EGFP*) zebrafish model. As a result, A549 cell proliferation and metastasis in the zebrafish xenograft model and angiogenesis in the transgenic zebrafish model were both found to be significantly inhibited by **1b** (Figure 6 and Figure 7). In this way, we demonstrated that TPP-targeted *α*-mangostin could induce apoptosis in A549 cancer cells both in vitro and in vivo.

## 4. Materials and Methods

### 4.1. General Procedures

A Bruker AV-400 spectrometer (Bruker, Fallanden, Switzerland) was used to obtain ^1^H and ^13^C NMR spectra (^1^H: 400 MHz, ^13^C: 100 MHz), with tetramethylsilane (TMS) as an internal reference at room temperature. High-resolution electrospray ionization mass spectra (HRESIMS) data were obtained on an IonSpec 7.0 T FTICR MS (IonSpec, Lake Forest, CA, USA). Column chromatography was performed with silica gel (Qingdao Haiyang Chemical Group Co., Ltd., Qingdao, China). *α*-Mangostin was isolated from the fruit hull of *Garcinia mangostana* in our previous study. Dulbecco’s modified Eagle’s medium (DMEM) and fetal bovine serum (FBS) were purchased from LabBiotech Co., Ltd. (Shandong, China). Celltracker CM-DiI was provided by Yeasen Biotechnology Co., Ltd. (Shanghai, China). Etoposide was offered by AdooQ BioScience (Nanjing, China). All other reagents were purchased from Tianjin Chemical Reagent Co. (Tianjin, China) and J&K Scientific Co. (Beijing, China).

### 4.2. General Procedure for the Synthesis of Compound ***1b***

#### 4.2.1. Synthesis of the Intermediate **1a**

Potassium carbonate (500 mg, 3.62 mmol) and 1,5-dibromopentane (1.5 mL, 11.01 mmol) were added to a solution of *α*-mangostin (300 mg, 0.73 mmol) in acetone (7.00 mL). The reaction mixture was stirred and refluxed at 65 °C until TLC detection indicated that the reaction was complete. The reaction mixture was extracted with ethyl acetate and then washed with saturated NaHCO_3_ (aq) and brine. The organic layer was dried over anhydrous sodium sulfate, concentrated in vacuo, and then purified via silica gel column chromatography with petroleum ether/ethyl acetate (100:0−100:10, *v*/*v*) to obtain an intermediate **1a** as a yellow solid (310 mg, 60%): ^1^H NMR (400 MHz, CDCl_3_): *δ*_H_ 13.42 (1H, s, -OH), 6.61 (1H, s), 6.19 (1H, s), 5.16 (2H, m), 4.05 (2H, d, *J* = 6.2 Hz), 3.99 (4H, m, -OCH_2_-), 3.73 (3H, s, -OCH_3_), 3.38 (4H, m, -CH_2_-Br), 3.27 (2H, d, *J* = 7.0 Hz), 1.90 (12H, m, -(CH_2_)_3_-), 1.77, 1.73, 1.60 (each, s, -CH_3_).

#### 4.2.2. Synthesis of Target Compound **1b**

Triphenylphosphine (250 mg, 0.95 mmol) was added to a solution of the intermediate **1a** (75 mg, 0.11 mmol) in toluene (3 mL). The mixture was then stirred at 80 °C for 12 h. The reaction residue was washed twice with 5 mL of toluene and dried in vacuo. The resulting oil was mixed with 10 mL of ether, sonicated for 1 h, and filtered to produce a cream-colored solid that was then purified by column chromatography (silica gel, dichloromethane/methanol, 100:0−100:25, *v*/*v*) to afford the target product **1b** (53 mg, 47%): ^1^H NMR (400 MHz, CDCl_3_): *δ*_H_ 13.48 (1H, s, -OH), 7.69−7.85 (15H, m, -(Ph)_3_-H), 6.70 (1H, s, Ph-H), 6.27 (1H, s, Ph-H), 5.23 (1H, t, *J* = 6.4 Hz), 5.16 (1H, t, *J* = 7.0 Hz), 4.08 (2H, d, *J* = 6.4 Hz), 4.01−4.08 (4H, m, -P-CH_2_-), 3.87 (4H, m, -OCH_2_-), 3.69 (3H, s, -OCH_3_-), 3.22 (2H, d, *J* = 7.0 Hz), 1.88−1.96 (12H, m, linker−CH_2_), 1.82, 1.72, 1.66, 1.62 (each, s, 3H, -CH_3_); ^13^C NMR (100 MHz, CDCl_3_): *δ*_C_ 181.9, 162.5, 159.5, 157.2, 155.2, 155.0, 143.8, 136.7, 135.1 (2C, d, *J*_c,p_ = 3.3 Hz, -P(Ph)_3_-C), 133.6 (d, *J*_c,p_ = 9.8 Hz, -P(Ph)_3_-C), 133.5 (d, *J*_c,p_ = 9.8 Hz, -P(Ph)_3_-C), 131.5, 131.2, 130.6 (d, *J*_c,p_ = 12.2 Hz, -P(Ph)_3_-C), 130.5 (d, *J*_c,p_ = 12.2 Hz, -P(Ph)_3_-C), 123.3, 122.4, 118.5 (d, *J*_c,p_ = 86.4 Hz, -P(Ph)_3_-C), 117.6 (d, *J*_c,p_ = 86.4 Hz, -P(Ph)_3_-C), 111.6, 110.0, 103.7, 99.0, 89.5, 68.5 (5-OCH_2_-), 67.9 (5′-OCH_2_-), 28.3 (4-CH_2_-), 28.2 (4′-CH_2_-), 27.0 (d, *J* = 17.0 Hz, 3-CH_2_-), 26.8(d, *J* = 17.0 Hz, 3′-CH_2_-), 26.1, 25.9, 25.8, 22.5 (d, *J* = 50.5 Hz, 1-CH_2_-), 22.4 (d, *J* = 50.5 Hz, 1′-CH_2_-), 22.2 (d, *J* = 4.0 Hz, 2, 2′-CH_2_-), 21.4, 18.2, 17.8; HRESIMS *m*/*z*: calcd for C_70_H_74_O_6_P_2_^2+^ [M − 2Br]^2+^ 536.2475; found, 536.2484.

### 4.3. Cell Culture

Four tumor cell lines (A549, K562, HepG2, and HeLa) were purchased from the Shanghai Institute for Biological Sciences, Chinese Academy of Sciences. All cell lines were cultured in Dulbecco’s modified Eagle medium (DMEM) containing 100 μg/mL streptomycin, 100 U/mL penicillin, and 10% fetal bovine serum (FBS) under humidified conditions with 5% CO_2_ at 37 °C.

### 4.4. In Vitro Cell Viability Assay

The MTT assay was used to assess the cytotoxic activities in vitro [32,33]. Tumor cells at the logarithmic growth stage were seeded in 96-well plates at a density of 1 × 10^4^ cells per well and incubated for 24 h at 37 °C. Then, the cells were treated for 48 h with various concentrations of the test compounds or a control. Following drug treatment, 20 μL of the MTT solution (5 mg/mL) was added to each well and incubated for 4 h. After incubation, the wells were centrifuged for 20 min at 3000 rpm at room temperature, the supernatant was discarded, and the formazan crystals were dissolved in DMSO (150 μL per well). All assays were carried out in triplicate. Additionally, the optical density OD value was measured at 492 nm using a microtiter plate reader (Thermo Fisher Scientific Inc., Waltham, MA, USA). The cell viability was determined using the following formula below:

Cell viability = (OD_s_ − OD_b_)/(OD_c_ − OD_b_) × 100% (1)

where the terms OD_s_, OD_b_, and OD_c_ represent the absorbance of the sample, blank, and negative control, respectively.

### 4.5. Cell Apoptosis Analysis by Flow Cytometry

Cellular apoptosis was detected using an Annexin V-FITC Apoptosis Detection Kit (Beyotime, C1062L) in line with the manufacturer’s instructions [13,34]. Cells were seeded in 12-well plates and incubated for 24 h. After 48 h of incubation with various concentrations of compound **1b** (2.25, 4.50, and 9.00 μM), cells were harvested. Next, the samples were diluted in 195 μL of binding buffer (Beyotime, Shanghai, China) after being washed with PBS. Then, the samples were incubated at room temperature in the dark with 10 μL of Annexin V-FITC and 5 μL of PI. Eventually, the attained cells were assessed via flow cytometry (BD Biosciences, San Jose, CA, USA). Flow cytometry data were analyzed using the FlowJo software (FlowJo LLC, Ashland, OR, USA).

### 4.6. Cell Cycle Arrest under Flow Cytometry

The cell cycle distribution of A549 cells exposed to compound **1b** was determined via flow cytometry [32,33]. A549 cells were seeded in 12-well plates (1 × 10^5^ cells/well) and treated with varying doses of compound **1b** (2.5, 5, and 10 μM). After 48 h of incubation, cells were harvested, washed twice with cold PBS, and suspended in 70% ice-cold ethanol at 4 °C overnight. The cells were then washed twice with cold PBS and stained at 37 °C for 30 min in the dark with a PI staining buffer supplemented with RNase (Beyotime, Shanghai, China). Thereafter, the cells were analyzed on a BD LSR Fortessa flow cytometer. ModFit LT software was used to process the data.

### 4.7. Detection of Intracellular Reactive Oxygen Species (ROS)

The levels of intracellular ROS were determined using the redox-sensitive dye 2′,7′-dichlorofluorescein diacetate (DCFH-DA) [17,35]. A549 cells were seeded in 12-well plates at a density of 1 × 10^5^ cells per well and incubated with compound **1b** at various concentrations (2.25, 4.5, and 9 μM) for 48 h. The cells were then washed thrice with a serum-free medium before being stained with DCFH-DA (10 μM) for 30 min at 37 °C in the dark. Cells were harvested after being washed with PBS one more time. The cells were analyzed using a BD LSR Fortessa flow cytometer. Data were obtained with the ModFit LT software.

### 4.8. Measurement of the Mitochondrial Membrane Potential (Δψ_m_)

The mitochondrial membrane potential was detected by staining with JC-1 (Beyotime, Nanjing, China). Cells were plated in 12-well plates (1 × 10^5^ cells per well) and treated with indicated doses of compound **1b** (5, 10, and 20 μM) for 48 h. After incubation, the cells were centrifuged for 3 min at 1000 rpm. Next, the supernatant was discarded, washed twice with a JC-1 staining buffer, and finally resuspended with 2 mL of a culture medium for flow cytometric measurement.

### 4.9. Western Blot Analysis

A549 cells were plated in 6-well plates (1 × 10^6^ cells/well) for 24 h. Then, cells were treated with compound **1b** at different concentrations (2 and 4 μM). After continuous incubation for 48 h, cells were washed twice with cold PBS and lysed using a lysis buffer including phenymethyl sulfonylfluoride (PMSF) and a freshly added protease inhibitor cocktail. Cells were then centrifuged for 10 min at 10,000 rpm, and the supernatant was collected to obtain the total proteins. A BCA protein assay kit (Beyotime, Shanghai, China) was used to measure the protein concentration in the supernatant. Protein samples (25 μg/lane) were separated via gel electrophoresis (SDS-PAGE) and electrotransferred to polyvinylidene difluoride (PVDF) membranes. The membranes were blocked with 5% skim milk in TBST for 2 h at room temperature, followed by overnight incubation at 4 °C with the corresponding primary antibodies. After washing with TBST, membranes were incubated with horseradish-peroxidase-conjugated secondary antibodies at room temperature for 1 h and then washed thrice with TBST. Lastly, the specific protein bands were detected using a hypersensitive enhanced chemiluminescence (ECL) kit (Beyotime, Shanghai, China). *β*-Actin was used as an internal control. Gel bands were quantified using the Image J software [36].

### 4.10. In Vivo Antitumor Activity Using Zebrafish Xenografts

Adult AB zebrafish were provided by Shanghai Feixi Biotechnology Co., Ltd. (Shanghai, China). Based on previous studies, a zebrafish tumor xenograft model was established in suitable embryos at 48 h post-fertilization (hpf) [37,38]. A549 cells were firstly labeled with CM-DiI (2 μM) and resuspended in an FBS-free medium to a density of 1 × 10^7^ cells/mL. In order to establish zebrafish xenografts, 5 nL of stained cells were microinjected into the yolk sac of 48 hpf anesthetized embryos, which were then incubated for 4 h at 28.5 °C. Following a 4-h incubation, tumor-bearing embryos were randomized into groups (15 embryos/group) and treated with different doses of compound **1b** (1, 3, and 9 μM) and a positive control etoposide (10 μM) for 48 h, respectively. At five days post fertilization (dpf), embryos were imaged via Leica confocal microscopy (Leica, Wetzlar, Germany). The density and focus number of red fluorescence were statistically analyzed using the Image J software.

### 4.11. In Vivo Angiogenesis Activity Using a Transgenic Zebrafish Model

A transgenic zebrafish *Tg* (*fli1:EGFP*) model was used to assess in vivo anti-angiogenesis activity, according to our previously reported method [38]. After breeding, embryos at 6 hpf were treated with the indicated concentrations of compound **1b** (0.3, 1, and 3 μM) and incubated for 48 h at 28.5 °C. Following incubation, the embryos were anesthetized with 0.02% tricaine. Confocal microscopy (Leica, Wetzlar, Germany) was used to observe the formation of intersegmental vessels (ISVs). The total average length of the ISVs was measured using Image J software (NIH, Bethesda, MD, USA). All animal procedures were approved by the Institutional Animal Care and Use Committee of Nankai University.

### 4.12. Statistical Analysis

Statistical analyses were performed using GraphPad Prism 6.0 (GraphPad, Software, San Diego, CA, USA). The data were presented as the mean ± SD (*n* = 3). The differences among the group means were assessed by a one-way analysis of variance (ANOVA) followed by Tukey’s multiple comparisons. A *p*-value less than 0.05 was considered statistically significant by ANOVA.

## 5. Conclusions

In this work, we designed and synthesized a novel xanthone to specifically target mitochondria. In recent years, natural products and their structural analogs have made significant contributions to pharmacotherapy, particularly in the treatment of cancer [39]. *α*-Mangostin is one of the most abundant natural xanthones with antioxidant and anticancer properties [10,11,12]. In this paper, the TPP-based modification of *α*-mangostin was carried out for the first time. Moreover, we demonstrated for the first time that mitochondrion-targeted *α*-mangostin can exert significant antitumor activities both in vitro and in vivo and could be further developed as a potential antitumor drug.

## Figures and Tables

**Table 1:** In vitro cytotoxic effects of compounds **1** and **1b** on four cancer cell lines.

Compounds	IC_50_ (μM) ^a^			
	A549	K562	HepG2	Hela
**1**	16.6 ± 0.6	25.3 ± 0.3	37.3 ± 0.8	27.5 ± 0.1
**1b**	3.0 ± 0.3	4.5 ± 0.5	5.0 ± 0.1	4.1 ± 0.1
Etoposide	27.4 ± 0.9	17.9 ± 1.0	21.0 ± 2.7	25.8 ± 2.0

^a^ The IC_50_ values are presented as the means ± standard deviations (*n* = 3). Cell viability was assessed as described in the biological assays section.

## Data Availability

Not applicable.

## Supplementary Materials

The following supporting information can be downloaded at: https://www.mdpi.com/article/10.3390/molecules28031016/s1, Fgure S1: 1H NMR (400 MHz, CDCl3) spectrum of compound **1**; Figure S2: 13C NMR (100 MHz, CDCl3) spectrum of compound **1**; Figure S3: 1H NMR (400 MHz, CDCl3) spectrum of compound **1a**; Figure S4: 1H NMR (400 MHz, CDCl3) spectrum of compound **1**; Figure S5: 13C NMR (100 MHz, CDCl3) spectrum of compound **1**; Figure S6: HRESIMS spectrum for compound **1b.**

## Sample Availability

Samples of the compounds are not available from the authors.
