# Supplementary material for: Design, Synthesis, Biological Evaluation, and Preliminary Mechanistic Study of a Novel Mitochondrial-Targeted Xanthone
Source: Molecules. 2023 Jan 19;28(3):1016. doi: 10.3390/molecules28031016 (PMC9920806; doi:10.3390/molecules28031016)
Supplement: Supplementary file 1 [file molecules-28-01016-s001.zip › molecules-2053872-supplementary.pdf]

Supplementary data for

Design, synthesis, biological evaluation, and preliminary mechanistic study of a novel mitochondrial-targeted xanthone

Contents

Figure S1 ^{1}H NMR (400 MHz, $CDCl_3$) spectrum of compound **1**

Figure S2 ^{13}C NMR (100 MHz, $CDCl_3$) spectrum of compound **1**

Figure S3 ^{1}H NMR (400 MHz, $CDCl_3$) spectrum of compound **1a**

Figure S4 ^{1}H NMR (400 MHz, $CDCl_3$) spectrum of compound **1b**

Figure S5 ^{13}C NMR (100 MHz, $CDCl_3$) spectrum of compound **1b**

Figure S6 HRESIMS spectrum for compound **1b**

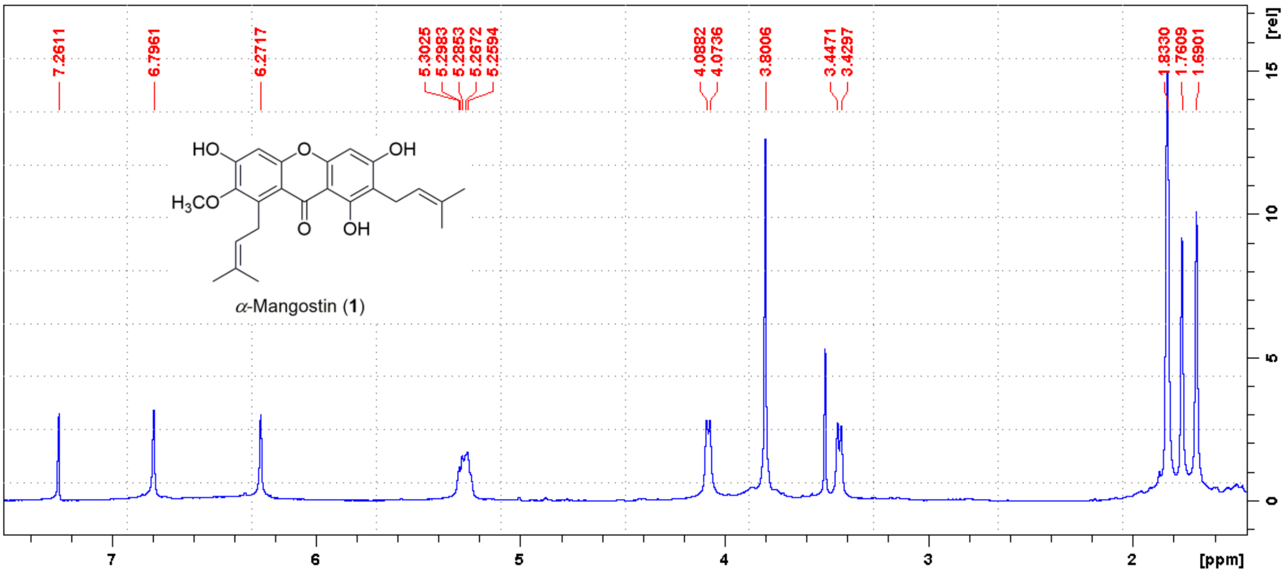

Figure S1 ^{1}H NMR (400 MHz, $CDCl_3$) spectrum of compound **1**

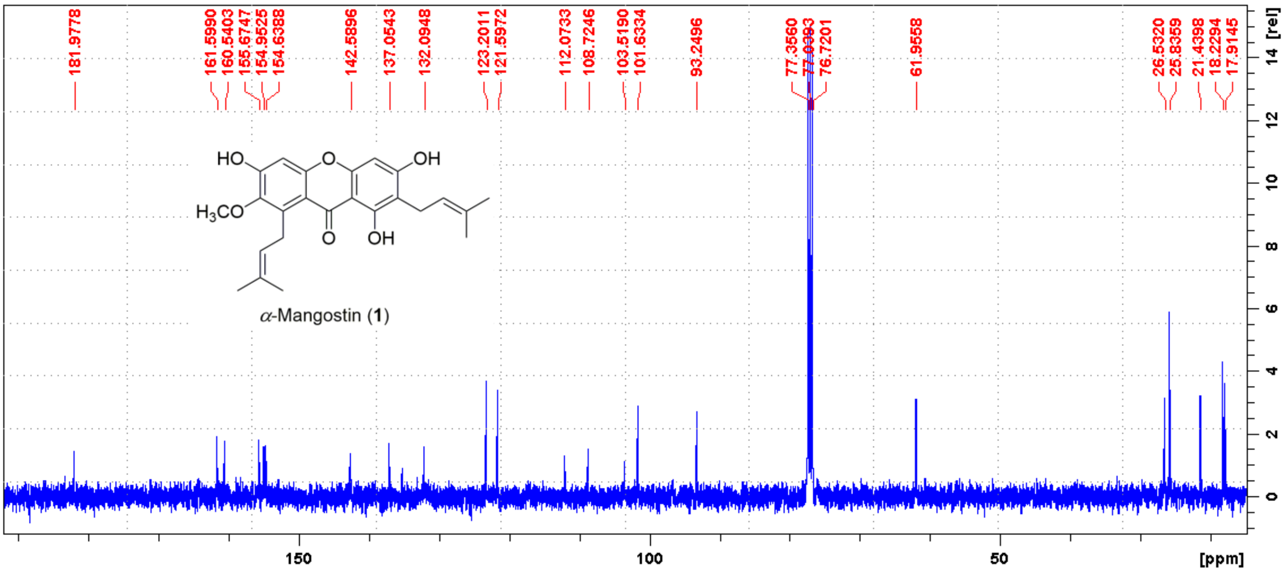

Figure S2 ^{13}C NMR (100 MHz, $CDCl_3$) spectrum of compound **1**

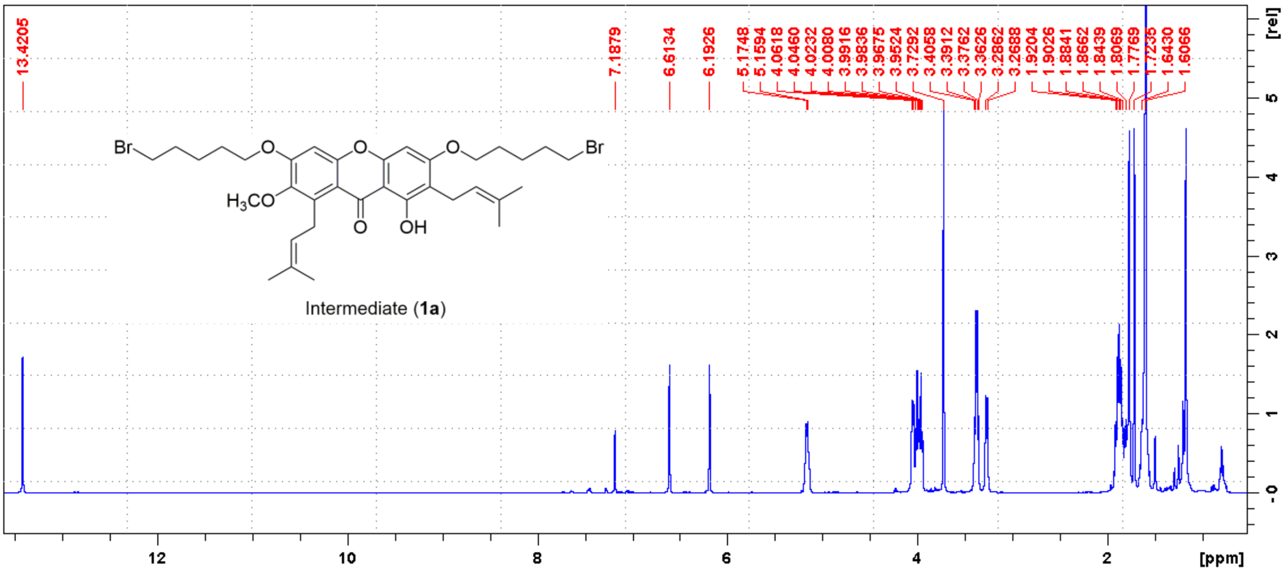

Figure S3 ^{1}H NMR (400 MHz, CDCl$_3$) spectrum of compound **1a**

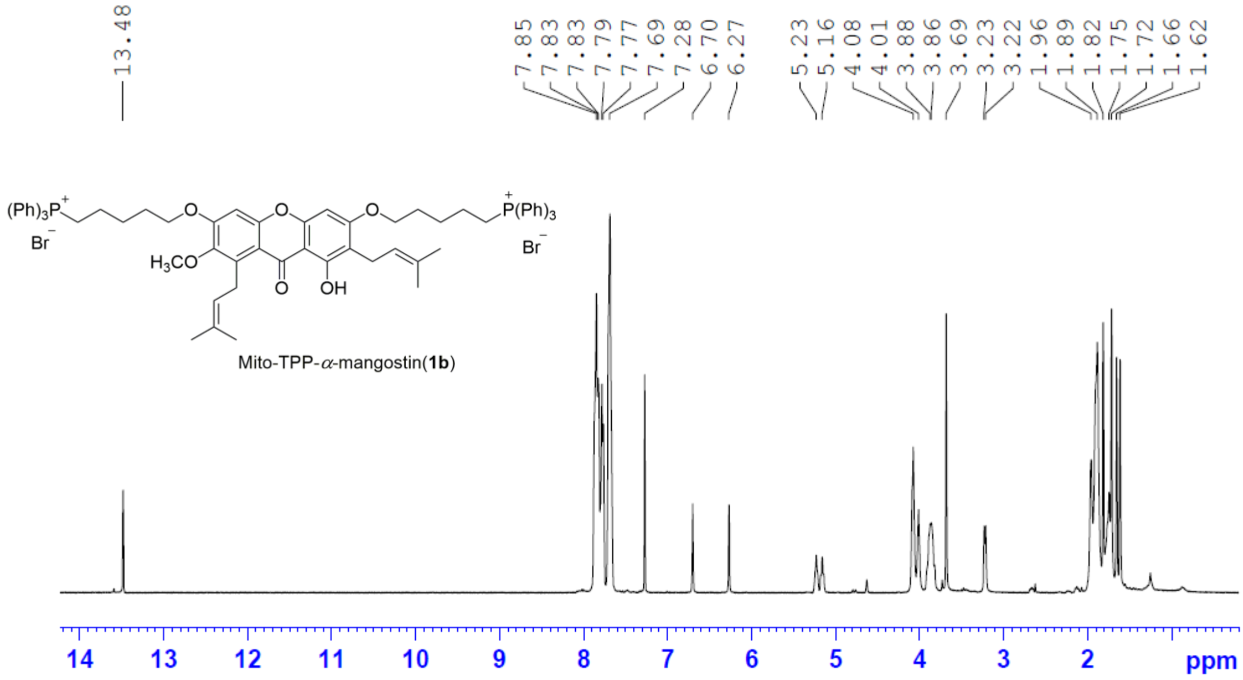

Figure S4 ^{1}H NMR (400 MHz, CDCl$_3$) spectrum of compound **1b**

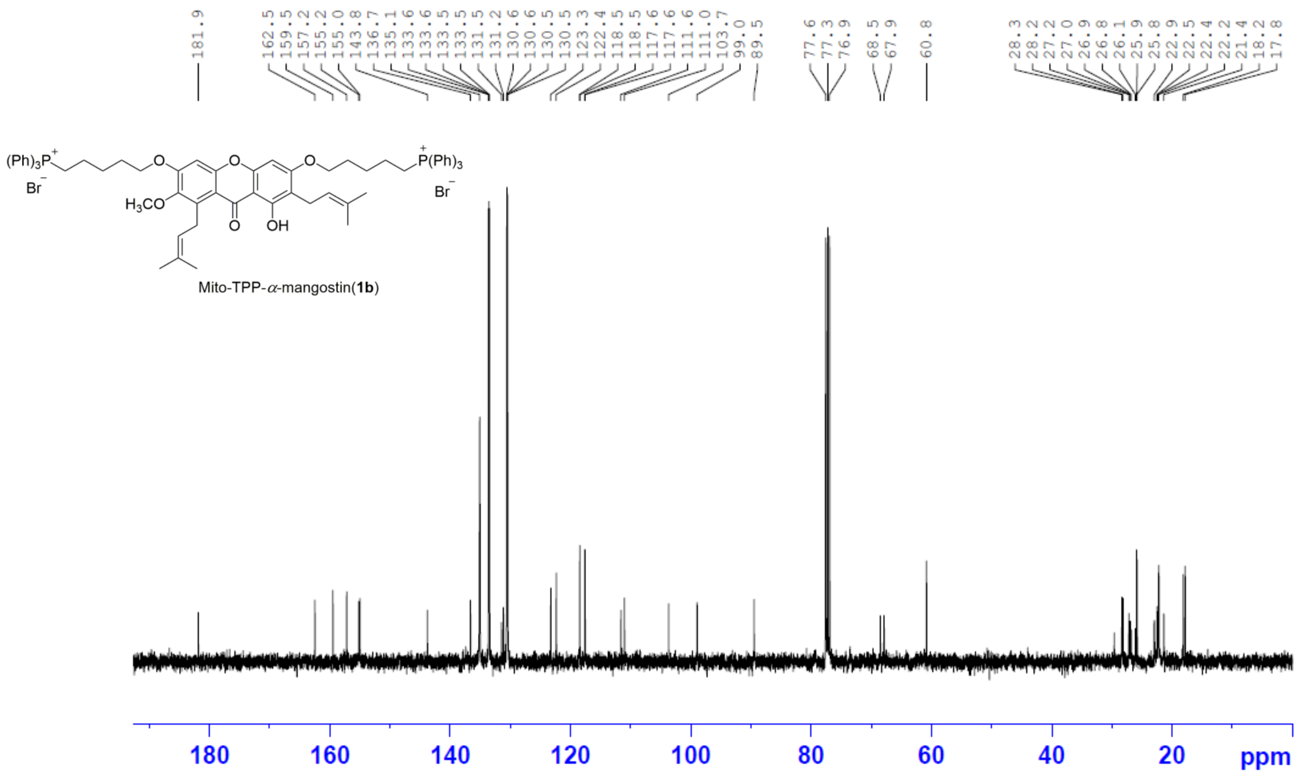

Figure S5 ^{13}C NMR (100 MHz, $CDCl_3$) spectrum of compound **1b**

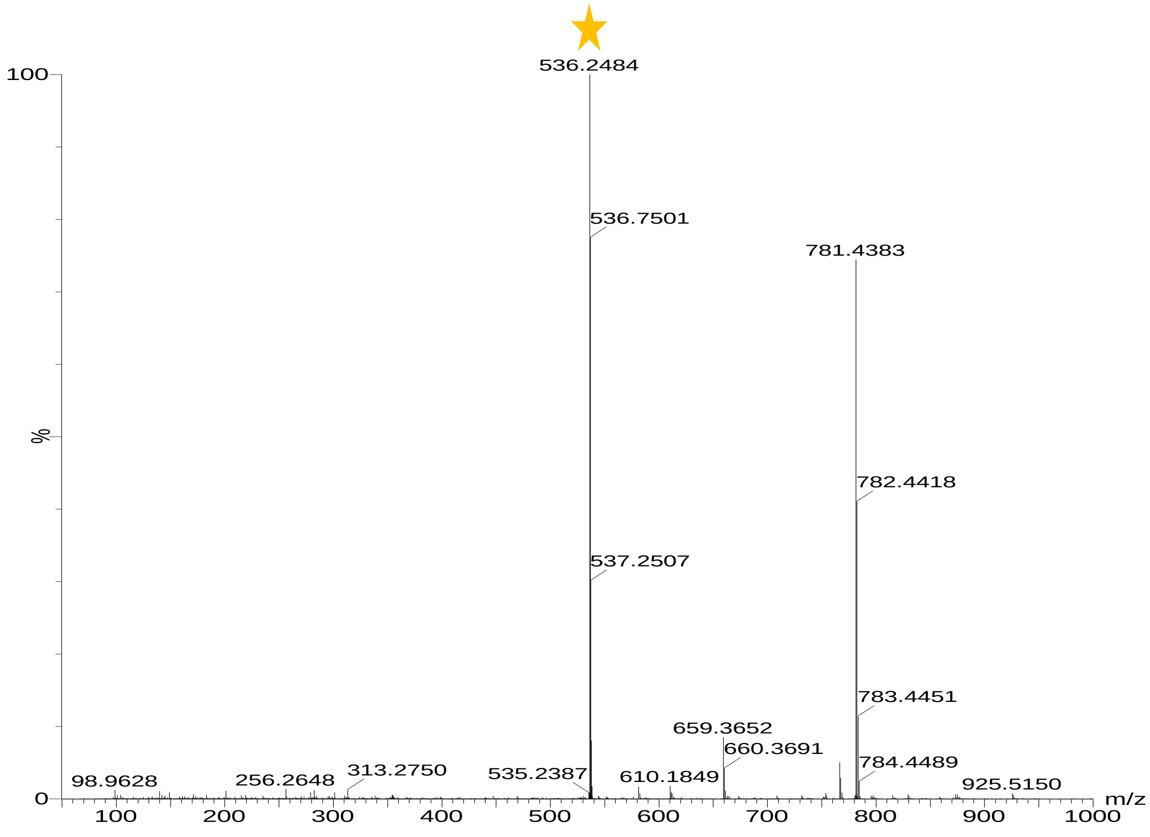

Figure S6 HRESIMS spectrum for compound **1b**
